# The effects of unilateral and bilateral eccentric overload training on hypertrophy, muscle power and COD performance, and its determinants, in team sport players

**DOI:** 10.1371/journal.pone.0193841

**Published:** 2018-03-28

**Authors:** Francisco Javier Núñez, Alfredo Santalla, Irene Carrasquila, Jose Antonio Asian, Jose Ignacio Reina, Luis Jesús Suarez-Arrones

**Affiliations:** 1 Department of Sports and Computing, Sport Faculty, Pablo de Olavide University of Sevilla, Sevilla, Spain; 2 Master de Futbol, Pablo de Olavide University of Sevilla, Sevilla, Spain; 3 Research Institute of Hospiltal 12 de Octubre (i+ 12), Madrid, Spain; 4 Dos Hermanas Radiological Center, Sevilla, Spain; Universidade de Tras-os-Montes e Alto Douro, PORTUGAL

## Abstract

The study aimed to compare the chronic eccentric-overload training effects of unilateral (lateral lunge) vs bilateral (half-squat) using an inertial device, on hypertrophy and physical performance. Twenty-seven young team sports male players performed a 4 sets of 7 repetitions of inertial eccentric overload training, biweekly for 6 weeks, distributed in unilateral lunge group (UG: age: 22.8 ± 2.9 years; body mass: 75.3 ± 8.8 kg; height: 177.3 ± 3.7 cm) and bilateral squat group (BG: age: 22.6 ± 2.7 years; body mass: 79.5 ± 12.8 kg; height: 164.2 ± 7 cm). Lower limb muscle volume, counter movement jump (CMJ), power with both (POWER), dominant (POWERd) and no-dominant leg (POWERnd), change of direction turn of 90° with dominant (COD90d) and no-dominant leg (COD90nd) and 180° (COD180d and COD180nd), and 10m sprint time (T-10m) were measured pre and post-intervention. The UG obtained an increase of *adductor major* (+11.1%) and *vastus medialis* (+12.6%) higher than BG. The BG obtained an increase of *vastus lateralis* (+9.9%) and *lateral gastrocnemius* (+9.1%) higher than UG. Both groups improved CMJ, POWER, POWERd, POWERnd, COD90 and DEC-COD90, without changes in T-10m. The UG decrease DEC-COD90nd (-21.1%) and BG increase POWER (+38.6%) substantially more than the other group. Six-weeks of unilateral / bilateral EO training induce substantial improvements in lower limbs muscle volume and functional performance, although unilateral training seems to be more effective in improving COD90 performance.

## Introduction

The majority of actions or movements produced in team sport matches require and increase the number of changes of direction (COD) at high speeds to be decisive in performance [1–3] or to have an advantage over opponents during competition [3, 4]. COD is a component of agility, and it describes a movement wherein no reaction to a stimulus is required and it is previously planned [2, 5]. COD ability is a multifactorial skill which, in its performance, depends on neuromuscular coordination [2], leg muscle strength and power [2, 3, 5] and straight sprinting speed [5–7]. During the COD, it is necessary to execute eccentric force rapidly to decelerate and develop the concentric strength to accelerate in the new direction [1, 2]. In depth, some authors have found that COD correlates with eccentric knee-flexor strength [8] and maximal eccentric lower-body strength [9, 10]. Despite those obvious physical requirements, there is a lack of information regarding the effects of eccentric-overload training on COD performance [2] and its influence on the different factors related to COD performance.

Resistance training produces muscular adaptations at both structural and functional levels [11–14]. Thus, numerous studies have demonstrated that chronic resistance exercise can produce an increase in hypertrophy and strength within the first 4 weeks of training [15, 16]. These adaptations are also shown in several studies that have used training protocols focusing on the concentric (CON) or eccentric (ECC) phases of the movement [12, 17–19]. Nevertheless, the increase in hypertrophy and strength is magnified using protocols in which the actions combine CON-ECC phases [12, 20, 21]. It is known that, in these CON-ECC actions, the capacity to produce force is much higher during the ECC than in the CON phase [22–27]. Therefore, some authors argue that training protocols, in which exercise is overloaded during the ECC phase of the movement, achieve greater strength gains than those in which the load is constant during both CON-ECC phases [27–29]. This has given rise to the concept of "eccentric overload" (EO).

Different training devices, using the inertia of rotating flywheel(s), have been designed to increase the EO during CON-ECC movements [30, 31]. This technology generates resistance by opposing the athlete’s effort with the inertial force generated by a lightweight rotating flywheel such that the same inertia must be overcome during each repetition by means of accommodated loading [32]. The training load on this technology can be regulated by increasing the speed of movement or by adding flywheel weights. Several studies have confirmed the efficacy of these training devices for improving hypertrophy [32–36], power [32, 34, 37], countermovement jump (CMJ) performance [9, 37–39], 10-m sprint time [4, 9, 38, 39] and COD [4, 9, 38–40].

In our knowledge, there are few studies that have investigated the effects of unilateral and bilateral strength training on physical performance (i.e., linear sprint, strength, power and COD) in team sport players [41, 42]. Only one study examined the effects of bilateral vs unilateral EO training using inertial devices on physical performance in team sports [4], and thus, the influence of this variable (bilateral vs unilateral EO using inertial devices) on the hypertrophy and COD performance factors is still unclear. Therefore, the aim of the present study was to compare the chronic eccentric-overload training effects of unilateral (lateral lunge) vs bilateral (half-squat) using an inertial device on hypertrophy and physical performance (measured as lower limb power, 10-m sprint, CMJ, COD).

## Materials and methods

### Subjects

Twenty-seven young healthy active males were divided in a unilateral lunge group (UG n = 14; age: 22.8 ± 2.9 years; body mass: 75.3 ± 8.8 kg; height: 177.3 ± 3.7 cm) and a bilateral squat group (BG n = 13; age: 22.6 ± 2.7 years; body mass: 79.5 ± 12.8 kg; height: 164.2 ± 7 cm), based on COD test results. All subjects were team sport players or sports science students and were actively training 3–4 times per week on average. All subjects were fully informed about the protocol and were required to give written consent in accordance with current national and international laws and regulations governing the use of human subjects (Declaration of Helsinki II) in research. The Institutional Ethics Committee (Pablo de Olavide University, Seville, Spain) approved this study.

### Experimental procedures

Magnetic resonance imaging (MRI) and muscle performance tests were measured in the pre- and post-experimental periods. All participants were familiarized with the test procedures to avoid any learning effects. On the 1^st^ day, a MRI was performed. On the 2nd day, subjects performed the field tests on an indoor futsal court in the following order: CMJ, 10-m sprint, and COD tests. There was a 5-min recovery period between the tests. Seventy-two hours later, lower limb power was assessed using an inertial device in the laboratory. Subjects were asked to abstain from performing any strenuous exercises on the day before each test, and they were also asked to follow a similar diet on the day of the test.

#### Magnetic resonance imaging

After one hour of supine rest to minimize the influence of a fluid shift on muscle size [43], axial scans were obtained using a 0.2 T MRI (E-scan, Esaote Biomedica, Genoa, Italy); Turbo 3-D T1; time to echo = 16 ms; time to repetition = 40 ms; NSA 1; scan time 3 min 57 s; field of view FOV 180 · 180 mm; matrix 256 · 256 pixels. For each participant, 20 continuous images of the thigh and calf with 6.3-mm-thick slices, with no spacing in between slices, were obtained. The cross-sectional area (CSA) of the *rectus femoris* (RF), the *vastus lateralis* (VL), the *vastus intermedius* (VI), the *vastus medialis* (VM), the *adductor major* (AM), the *lateral gastrocnemius* (LG), the *medial gastrocnemius* (MG), and the *soleus* (S) muscles were analysed in the dominant leg.

Three images were selected for each subject. The first was at the insertion of the AM to analyse the volume of each quadricep muscle. The second was at the junction of the popliteal artery with the fascia of the adductor major to analyse the volume of the AM. The third was coinciding with the section of the greater perimeter of the leg to analyse the volume of LG, MG, P and S. Every image was analysed by manually encircling the individual muscles using OsiriX dicom viewer 2.2.1 software (Osirix Foundation, Ginebra, Suiza). All muscles were encircled separately, but for the analysis, VL and VI appeared together. Subjects also refrained from excessive muscular exercises for 72 h before reporting to the MRI facility.

#### Field tests

After a 7- to 8-min standardized general warm-up, which included jogging, joint mobility exercises, and 2 sets of 10 repetitions of squats, field tests were carried out. Additionally, a specific warm-up was performed before each test: 1 set of 5 reps (CMJ) and 2 sub-maximal efforts (linear sprint and COD).

Countermovement jump test (CMJ). The CMJ vertical jump test was used to maximize the stretch-shortening cycle activity and assess the explosive strength of the lower limb muscles. The test was performed using an infrared contact platform (Optojump®, Microgate, Bolzano, Italy). During the CMJ, all subjects were instructed to rest their hands on their hips, which was followed by a vertical jump at maximal effort and landing in an upright position keeping their knees extended. The CMJ was performed three times, and it was separated by 45 s of passive recovery. The best jump performance was used for the subsequent statistical analysis.

10-m linear sprint test. Subjects performed a 10-m sprint test, and time was recorded using photoelectric cells (Racetime2, Microgate®, Bolzano, Italy). The front foot was placed 0.5 m behind the first timing gate, and players started voluntarily so the reaction time was eliminated. After a specific warm-up, including the 2 submaximal efforts, two trials were completed. Two minutes of passive recovery occurred between the trials. The best performance trial was used for the subsequent statistical analysis (T-10m).

COD test. COD capacity was measured by four tests. On the one hand, subjects performed two maximum 5 m+5 m straight line sprints with a COD turn of 90°. One of them was done with the dominant leg on the outside (COD90d) during the turn, and the other with the non-dominant leg (COD90nd). The turning space was fixed by 4 sticks (height: 1.5 m) which were placed vertically in other to avoid a curvilinear trajectory. On the other, subjects performed two maximum 5 m+5 m straight line sprints with a COD turn of 180°. Subjects performed the COD by touching a line with the dominant leg (COD180d) and with the non-dominant leg (COD180nd). Subjects were encouraged to complete two trials of each test as fast as possible. Two minutes of passive recovery occurred between trials and tests. The time was recorded with the same photoelectric cells (Racetime^2^, Microgate^®,^ Bolzano, Italy), using two of them in COD90d and COD90nd (placed on the start and finish line) and only one in the start/finish line of the COD180d and COD180nd. As in the 10-m test, the front foot was placed 0.5 m before the first timing gate, and subjects started voluntarily so the reaction time was eliminated. The best time of each test (COD90d, COD90nd, COD180d and COD180nd) was compared with the T-10m to estimate the percentage mean of speed loss due to executing the COD (DEC-COD90d, DEC-COD90nd, DEC-COD180d and DEC-COD180nd), through the formula [(COD–T-10m)/ T-10m) x 100].

#### Laboratory test

After a 7- to 8-min standardized general warm-up, included jogging, joint mobility exercises and two sub-maximal sets of 8 reps of a half-squat exercise, two lower limb power tests were done. The tests consisted of the assessment of power in the half-squat and lateral lunge exercises using a non-gravity dependent flywheel inertial device (Exxentric kBox, Exxentrix AB, Stockholm, Sweden), allowing subjects to perform maximal CON and ECC actions [44]. Subjects performed 2 monitored sets of all-out 8 repetitions of squats (0.10 kg/m^2^ moment inertia) with both legs (POWER) and a lateral lunge (0.05 kg/m^2^ moment inertia) with dominant (POWERd) and non-dominant leg (POWERnd). The best of the two sets, according to the criteria of higher rotational mean power relative to body weight, was considered for subsequent analysis. To produce EO, subjects were requested to push maximally through the entire range of motion of the CON action and then gently resist during the initial part (first 20°-30°) of the subsequent ECC action before resisting maximally, aiming at bringing the wheels to a stop at ~90° knee angle before initiating the next cycle [45]. There was a 2-min rest in the standing position between each set. As in previous studies with these inertial devices [37], power and velocity during the training session were sampled at 100 Hz using a rotatory encoder (SmartCoach^TM,^ SmartCoach Europe AB, Stockholm, Sweden) and associated software (SmartCoach^®^ v.5.2.0.5).

### Flywheel resistance exercise training

Participants underwent a 6-week training programme based on bilateral squat (BG) or unilateral lunge (UG) on a flywheel inertial device (Exxentric kBox, Exxentrix AB, Stockholm, Sweden). They performed 2 sessions/week with at least 48 h of rest between the sessions in the same conditions of the test. Each session was structured in a brief standardized warm-up (like that used before in the power test) and 4 sets of 7 repetitions of squat (0.10 kg/m^2^ moment inertia) in BG and a unilateral squat with each leg (0.05 kg/m^2^ moment inertia) in UG. A 3-min recovery period was allowed between sets.

### Statistical analysis

Data are presented as mean ± SD. All data were log-transformed for analysis to reduce bias arising from non-uniformity error and then analysed for practical significance using magnitude-based inferences [46]. The effect size (ES, 90% confidence limit (CL)) in the selected variables was calculated using the SD. Threshold values for Cohen ES statistics were >0.2 (small), >0.6 (moderate), and >1.2 (large) [46]. The chance that any difference was better/greater (i.e., greater than the smallest worthwhile change, SWC [0.2 multiplied by the between-subject standard deviation, based on Cohen’s d principle, ES]) or similar or worse/smaller than the other group, was subsequently calculated. Quantitative chances of beneficial/better or detrimental/poorer effect were assessed qualitatively as follows [46, 47]: <75%, unclear; >75–95%, likely; >95–99%, very likely; and >99%, almost certain. If the chance of having beneficial/better or detrimental/poorer was >75%, the true difference was considered clear (substantial)[48].

## Results

The pretreatment MRI analysis showed that the muscle volume of BG was greater in AM (+8.9% [90%CL: -18.21;1.28] moderate ES) and S (+17.1% [90%CL: -25.12;8.31], large ES) than in UG. Likewise, the pretreatment field analysis showed that the DEC-COD90d (+15.4% [90%CL: -6.72;42.62], moderate ES), the DEC-COD90nd (+20.3% [90%CL: -4.4;51.44], moderate ES), the DEC-COD180d (+12.7% [90%CL: -4.85;33.59], moderate ES), and the DEC-COD180nd (+15.1% [90%CL: -3.48;37.34], moderate ES) of UG were greater than in BG.

**Fig 1:**
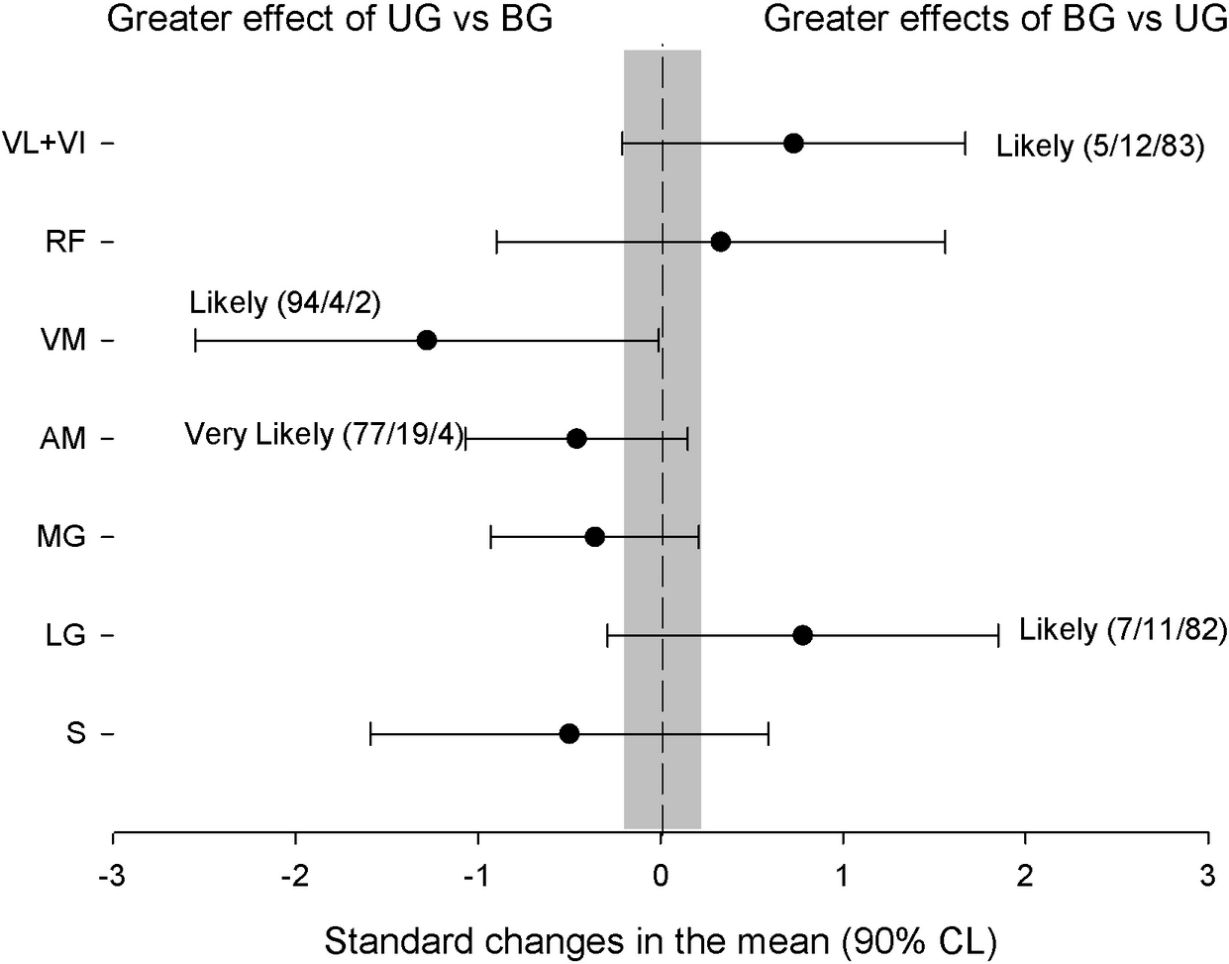
Comparison of the training effects produced in the experimental groups for CSA values.

The pre- and post-analysis of lower-limb MRI and tests of UG and BG are shown in Tables 1 and 2, respectively. Both training groups substantially increased VM and MG muscle volumes (Tables 1 and 2). The UG obtained a greater increased in AM (ES = 0.46 [90%CL: -0.15;1.7], small) and VM (ES = 1.28 [90%CL: 0.15;2.4], large) than BG (Fig 1). The BG substantially increased the muscle volume of the VL + VI (ES = 0.73 [90%CL: -0.21;1.67], moderate) and LG (ES = 0.78 [90%CL: -0.3;1.85], moderate) compared with UG (Fig 1).

**Table 1:** Relative differences and qualitative outcomes in cross-sectional area (CSA), functional test post a unilateral squat training period. Data are mean ± SD.

Variable	Pre	Post	*% Diff (CL)*	*ES (CL)*	Chances %	Qualitative magnitude
*MRI*						
VL+VI	42.16 ± 10.46	43.71 ± 9.75	4.34 (-4.89; 14.46)	0.16 (-0.19; 0.52)	43/53/5	Unclear
RF	7.24 ± 1.86	7.20 ± 2.19	-1.91 (-11,91; 9,22)	-0.07 (-0.47; 0.33)	12/59/29	Unclear
VM	17.14 ± 4.09	19.31 ± 5.14	12.66 (6.35; 18.97)	0,41 (0.21; 0.61)	95/5/0	Very Likely
AM	18.06 ± 3.02	20.08 ± 3.23	11.13 (4.86; 17.77)	0.61 (0.27; 0.95)	98/2/0	Very Likely
MG	15.48 ± 4.17	16.70 ± 4.25	8.22 (3.60; 13.05)	0.27 (0.12; 0.42)	79/21/0	Likely
LG	10.35 ± 2.43	10.43 ± 2.47	0.53 (-9.56; 11.75)	0.02 (-0.41; 0.45)	24/58/19	Unclear
S	28.12 ± 3.84	29.22 ± 4.07	3.81 (-0.42; 8.21)	0,26 (-0.03; 0.56)	65/34/1	Unclear
*Field Test*						
CMJ (cm)	34.42 ± 4,91	35.98 ± 4,89	4.69 (1,76; 7,70)	0.28 (0.11; 0.45)	78/22/0	Likely
T-10m (s)	1.79 ± 0.19	1,85 ± 0.08	4.14 (-2.91; 11.71)	0.31 (-0.22; 0.84)	64/30/6	Unclear
COD180d (s)	2.70 ± 0.11	2.66 ± 0.12	-1.22 (-2.40; -0.02)	-0.29 (-0.57; -0.01)	0/29/71	Unclear
COD180nd (s)	2.68 ± 0.12	2.64 ± 0.14	-1.53 (-3.35; 0.33)	-0.33 (-0.74; 0.07)	2/26/72	Unclear
COD90d (s)	2.54 ± 0.13	2.44 ± 0.15	-4.04 (-6.74; -1.27)	-0.75 (-1.27; -0.23)	0/4/96	Very likely
COD90nd (s)	2.55 ± 0.16	2.46 ± 0.16	-3.60 (-5.53; -1.65)	-0.54 (-0.83; -0.24)	0/3/97	Very Likely
DEC-COD180d (%)	53.11 ± 23.70	44.08 ± 6.60	-12.93 (-26.56; 3.21)	-0.41 (-0.91; 0.09)	3/21/76	Likely
DEC-COD180nd (%)	52.25 ± 23.71	42.84 ± 7.76	-14.10 (-29.44; 4.57)	-0.43 (-0.99; 0.13)	3/20/76	Likely
DEC-COD90d (%)	44.86 ± 25.67	32.12 ± 7.09	-23.12 (-38.35; -4.14)	-0.60 (-1.11; -0.10)	1/8/91	Likely
DEC-COD90nd (%)	45.41 ± 26.92	33.10 ± 7.53	-21.16 (-34.98; -4.40)	-0.51 (-0.92; -0.10)	0/10/90	Likely
*Laboratory Test*						
POWER (w/kg)	6.82 ± 1.52	8.14 ± 2.10	18.58 (9.02; 28.96)	0.70 (0.36; 1.05)	99/1/0	Very Likely
POWERd (w/kg)	5.35 ± 1.37	7.70 ± 1.65	44.83 (27.96; 63.92)	1.44 (0.96; 1.92)	100/0/0	Almost Certainly
POWERnd (w/kg)	5.48 ± 1.12	7.46 ± 1.75	35.45 (20.64; 52.07)	1.42 (0.88; 1.97)	100/0/0	Almost Certainly

**NOTE:** VL+VI = Vastus lateralis and vastus intermedius; RF = rectus femoris; VM = vastus medialis; AM = adductor major; LG = lateral gastrocnemius; MG = medial gastrocnemius; P = Peroneus; S = soleus; CMJ = Couter movement junp; T-10m = time in 10 m linear sprint test; COD180d and COD180nd = time in 5+5 m sprint change of direction of 180° with dominant and non-dominat foot; COD90d and COD90nd = time in 5+5 m sprint change of direction of 90° with dominant and non-dominat foot; DEC-COD = percentage mean speed loss due to execute CODs compared to 10 m sprint time; POWER, POWERd and POWERnd = power relative to bodyweight in squat exercise with both legs, dominant leg and non-dominant leg. CL = confidence limits; ES = effect size; Chances = percentage chance of having better/similar/poorer values.

**Table 2:** Relative differences and qualitative outcomes in cross-sectional area (CSA) and functional test post a bilateral squat training period. Data are mean ± SD.

Variable	Pre	Post	*% Diff (CL)*	*ES (CL)*	Chances %	Qualitative magnitude
*MRI*						
VL+VI	43.88 ± 5.67	48.62 ± 8.84	9.98 (3.35; 17.04)	0.62 (0.21; 1.02)	96/4/0	Very Likely
RF	8.17 ± 2.27	8.63 ± 2.14	6.47 (-1.69; 15.31)	0.21 (-0.06; 0.48)	53/46/1	Unclear
VM	18.90 ± 4.40	21.03 ± 4.01	11.26 (2.02; 20.05)	0.46 (0.13; 0.78)	91/9/0	Likely
AM	19.84 ± 3.14	20.56 ± 3.83	3.10 (-1.86; 8.32)	0.18 (-0.11; 0.46)	44/54/2	Unclear
MG	16.59 ± 2.92	17.69 ± 2.85	7.02 (2.17; 12.10)	0.34 (0.11; 0.58)	85/15/0	Likely
LG	9.26 ± 2.06	10.17 ± 2.78	9.09 (-0.04; 19.05)	0.33 (0.00; 0.67)	75/24/1	Likely
S	34.11 ± 5.72	33.47 ± 6.10	-2.07 (-4.75; 0.69)	-0.12 (-0.27; 0.04)	0/83/17	Unclear
*Field Test*						
CMJ (cm)	34.65 ± 3.78	36.52 ± 4.89	5.12 (1.35; 9.03)	0.42 (0.11; 0.73)	89/11/0	Likely
T-10m (s)	1.85 ± 0.07	1.85 ± 0.09	0.03 (-1.76; 1.85)	0.01 (-0.44; 0.45)	23/56/21	Unclear
COD180d (s)	2.68 ± 0.15	2.66 ± 0.14	-0.66 (-3.54; 2.30)	-0.11 (-0.62; 0.39)	15/47/39	Unclear
COD180nd (s)	2.65 ± 0.15	2.65 ± 0.14	-0.06 (-1.38; 1.28)	-0.01 (-0.23; 0.21)	6/86/8	Unclear
COD90d (s)	2.51 ± 0.11	2.43 ± 0.12	-3.21 (-4.57; -1.84)	-0.70 (-1.00; -0.40)	0/1/99	Very Likely
COD90nd (s)	2.49 ± 0.13	2.45 ± 0.14	-1.63 (-4.12; 0.92)	-0.29 (-0.74; 0.16)	4/33/64	Unclear
DEC-COD180d (%)	44.94 ± 6.93	43.94 ± 6.78	-2.23 (-12.28; 8.97)	-0.14 (-0.81; 0.53)	19/37/44	Unclear
DEC-COD180nd (%)	43.13 ± 6.22	42.95 ± 4.86	0.09 (-6.48; 7.13)	0.01 (-0.40; 0.41)	21/60/19	Unclear
DEC-COD90d (%)	35.77 ± 5.29	31.46 ± 7.24	-13.17 (-20.91; -4.66)	-0.84 (-1.39; -0.28)	0/3/97	Very Likely
DEC-COD90nd (%)	34.53 ± 6.48	32.29 ± 6.34	-6.61 (-16.38; 4.30)	-0.34 (-0.89; 0.21)	5/28/67	Unclear
*Laboratory Test*						
POWER (w/kg)	6.61 ± 1,58	9.05 ± 1,70	38.63 (29,17; 48,78)	1.15 (0.90; 1.40)	100/0/0	Almost Certainly
POWERd (w/kg)	4.85 ± 1.13	7.35 ± 1.36	53.23 (37.29; 71.01)	1.55 (1.15; 1.94)	100/0/0	Almost Certainly
POWERnd (w/kg)	5.00 ± 1.15	6.95 ± 1.34	40.68 (26.09; 56.95)	1.24 (0.84; 1.64)	100/0/0	Almost Certainly

**NOTE:** VL+VI = Vastus lateralis and vastus intermedius; RF = rectus femoris; VM = vastus medialis; AM = adductor major; LG = lateral gastrocnemius; MG = medial gastrocnemius; P = Peroneus; S = soleus; CMJ = Couter movement junp; T-10m = time in 10 m linear sprint test; COD180d and COD180nd = time in 5+5 m sprint change of direction of 180° with dominant and non-dominat foot; COD90d and COD90nd = time in 5+5 m sprint change of direction of 90° with dominant and non-dominat foot; DEC-COD = percentage mean speed loss due to execute CODs compared to 10 m sprint time; POWER, POWERd and POWERnd = power relative to bodyweight in squat exercise with both legs, dominant leg and non-dominant leg. CL = confidence limits; ES = effect size; Chances = percentage chance of having better/similar/poorer values.

**Fig 2:**
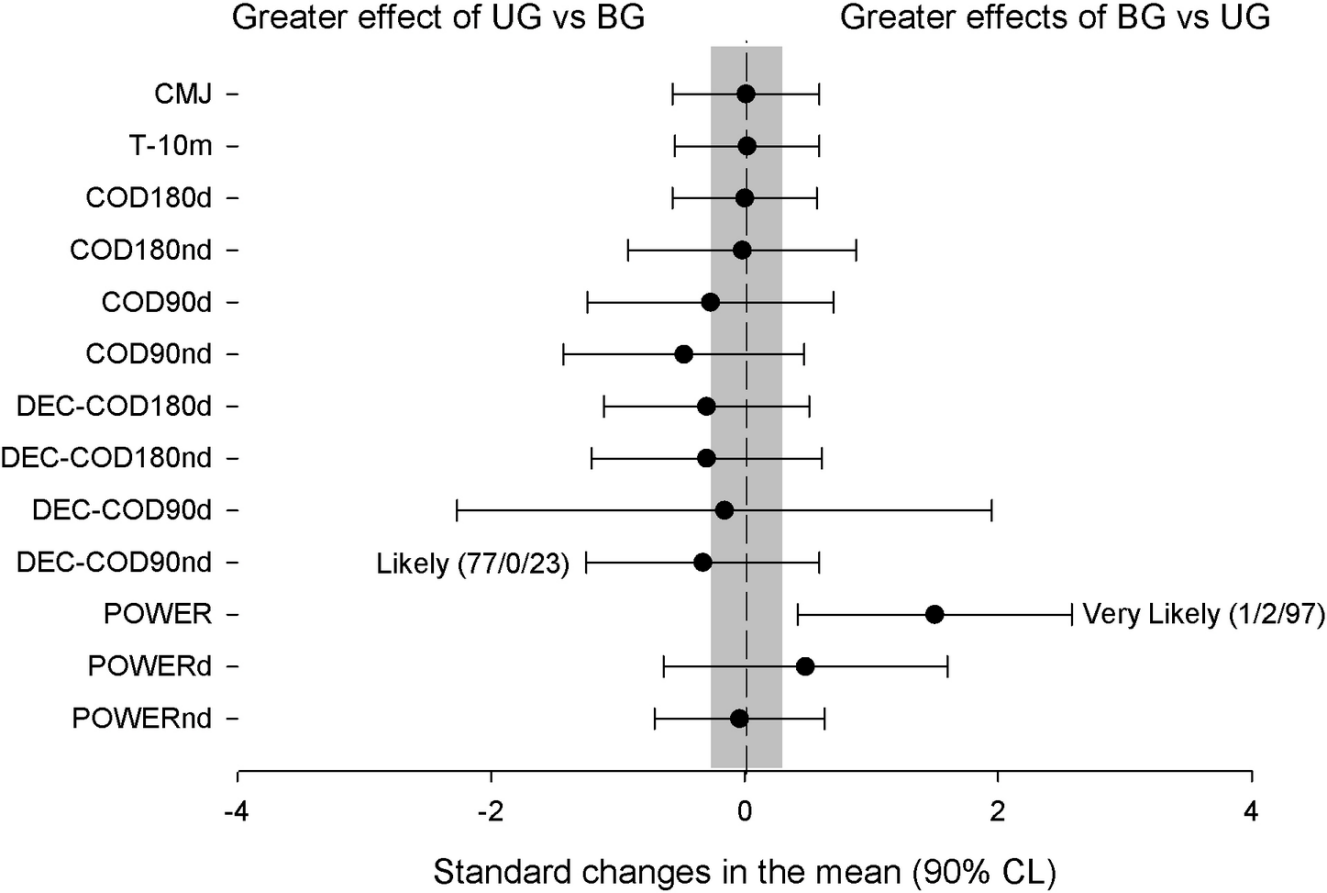
Comparison of the training effects produced in the experimental groups for functional test.

In a field test, both training groups substantially increased the CMJ and substantially decreased COD90d and DEC-COD90d without substantial differences between groups (Fig 2). The UG substantially decreased DEC-COD90nd, obtaining substantial differences compared with BG (ES = 0.33 [90%CL: -0.59;1.25], small). Likewise, the UG substantially decreased DEC-COD180d and DEC-COD180Nd, without substantial differences compared with the BG. There was no substantial effect of training in T-10m for each group (Tables 1 and 2), although the UG increased, while the BG did not change T-10m.

In a laboratory test, both groups substantially increased the POWER, POWERd and POWERnd. The increased of POWER in BG (ES = 1.5 [90%CL: -0.42;2.58], moderate) was greater than in UG (Fig 2).

## Discussion

The aim of the present study was to compare the effects of chronic eccentric-overload training of unilateral (lateral lunge) vs bilateral (half-squat) using an inertial device in muscle volume, CODs, power, CMJ and 10-m sprint improvements in team sports players. The main findings of this study were as follows. 1. The UG training increased AM and VM muscle volumes substantially more than did BG, while BG increased in VL+VI and LG muscle volume substantially more than did UG. 2. Both groups decreased the time in COD90d, but the UG training provided a better COD 90° transition phase in the non-dominant leg than did BG training; 3. Both types of training increased the power generated in the inertial system during the half-squat and unilateral lunge, but the BG increment was substantially higher than UG in the half-squat test. 4. Both types of training increased the CMJ performance but did not change the time in 10-m sprint test.

Inertial devices have been shown to be very effective in improving muscle size in short-term strength training periods [49]. In our study, using a flywheel inertial device for only 6 weeks, muscle volume increased from 7–8% in MG to 11–12% in MV in both training groups. Only the UG led to an increase in muscle volume of the AM (+ 11%) being substantially greater than that obtained by BG. Likewise, only the BG increased the VL + VI by 10% and LG by 9%, obtaining substantial differences compared with the UG. Previous studies [32, 34–36] using knee extension flywheel inertial devices during similar training periods (5 weeks) have shown similar improvements in the same muscles, but in smaller magnitudes, VM (from 7.9% to 8.6%) and VL + VI (From 5.8% to 7.8%). Furthermore, these studies have also described increases in the *rectus femoris* volume in both unilateral (From 9.1% to 9.9%, [32, 34, 36]), and bilateral exercises (11.4%, [35]), which did not occur in our study. This finding may be due, at least in part, to the different mechanical demands on the quadricep’s musculature. With an open kinetic chain exercise (i.e., using a knee extension flywheel inertial device), in which there is a block of hip movement, there seems to be a more pronounced increase in the RF [32, 34–36]. Nevertheless, in closed kinetic chain exercises (i.e., using a Squat flywheel inertial device), in which there is a hip extension, there seems to be a more pronounced increase in the lateral-medial musculature of the quadriceps [50]. This effect of muscular demand can be appreciated even in the same inertial device by analysing the UG and BG training. In our study, there was similar muscular growth in all muscles except in the AM, which increased in UG that performed the lateral lunge exercise. By using 3-D musculoskeletal dynamics simulation models to compare the joint reaction forces, it is known that it is there is a greater demand of the horizontal force in the lateral lunge than in the squat [51]. Furthermore, Fisher and Wallin [52] argued that the use of unilateral strength training can increase the force production of the stabilizing muscles (i.e., hip adductors). These two aspects could partly explain the AM increment in the UG group.

In exploring the relationship between muscle volume and speed, Chelli et al [53] described significant and positive correlations between increased thigh muscle hypertrophy and 5-m sprint speed (r = 0.48) in junior soccer players. However, despite the thigh muscle size increase found in our results, the BG and UG T-10m did not change. Our results are in agreement with those obtained by De Hoyo et al. [38], that reported no improvements in a 10-m sprint after 10 weeks of training using the same bilateral squat exercise in a similar flywheel inertial device. Likewise, Tous-Fajardo et al [39] obtained similar results after 11 weeks of combined unilateral strength training (including a lateral lunge inertial device). Nevertheless, Gonzalo-Skok et al [4] recently compared unidirectional-bilateral vs multidirectional-unilateral exercises using an inertial device and showed that both types of training-induced substantial enhancements in short distance sprints. The different moment inertia (from 0.05 to 0.1 vs 0.27 kg/m^2^), different devices (K-Box vs conical-pulley) and how they were used could explain these differences. In fact, these authors considered that it was very unlikely to obtain EO with a conical-pulley inertial device; moreover, in more stable exercises (i.e., Squat Kbox vs. Squat conical-pulley), a more consistent EO can be developed [4]. Given this controversy caused by both factors, EO and stability, together with the fact that a recent meta-analysis has described that an increase in force is significantly higher with the existence of EO during the exercise [49], it could be interesting to study EO during exercise for the improvement of velocity in short distance sprints.

This disparity of results about the effects of UG/BG training on sprint speed is also seen in the COD performance. It has recently been suggested that unilateral and bilateral training may be equally efficacious in improving COD [41]. Nevertheless, some studies have shown substantially greater COD performance improvements after unilateral training using conventional [42, 52] and inertial devices [4]. COD ability is a multifactorial skill in which performance depends on neuromuscular coordination [2], leg muscle strength and power [2, 3, 5] and a straight sprinting speed [5–7]. Regarding the importance of each factor, studies focused on improving COD performance with unilateral/bilateral training do not differentiate if the COD can be improved by increasing all these factors or just by increasing only the straight sprinting speed. We analysed the percentage of speed lost due to execute the COD (DEC-COD) just to differentiate the influence of these factor in the increase of COD performance after training.

In our study, the UG substantially improved both COD90d and COD90nd and all DEC-CODs (90° and 180°), while BG improved only COD90d and DEC-COD90d. The magnitude of these changes in COD90 (ES 0.41 to 0.84) was similar to that recently reported in COD turns of 45° after either unilateral or bilateral training using an inertial device [4]. As in other studies [4, 42], UG always obtained more robust adaptations in all COD tests (i.e., greater mean ES) than BG (Fig 2), but there was only a substantial interaction between groups for DEC-COD90nd. In both our study and in Gonzalo-Skok et al. [42], UG obtained better results in the non-dominant leg than BG. These authors argued there was a difference between dominant and non-dominant vertical ground force that favoured the preferred leg during a bilateral squat. It could be possible that BG might exert more effort with the dominant leg (vs non-dominant leg), whilst UG had made the same effort with the dominant and non-dominant leg. As the sprint capacity did not change in our study, COD improvements could depend on good neuromuscular coordination plus muscle strength and power to stop the previous inertia and to accelerate later in the new direction [2]. This effect seems to be reflected in the lower loss of time to stop the previous inertia during the COD90 in both groups (DEC-COD90). It is known that these adaptations depend on the weekly eccentric-overload stimulus elicited by Tous et al [39]. Thus, the fact that BG only improved DEC-COD90d could be partly explained by a different EO stimulation on the dominant and non-dominant leg (as explained before). In this respect, the absence of an electromyographic registry during the training sessions to confirm this suggestion as a limitation of our study.

The CMJ performance was increased from 4.7 to 5.1% (for UG and BG, respectively) without an inter-group effect. This change’s magnitude and the lack of inter-group effect were similar to those recently reported by Gonzalo-Skok et al. [4]. However, BG appears to have a higher effect on jumping performance than unilateral training (ES-UG = 0.28; ES-BG = 0.42). Although both exercises improved POWER, POWERd and POWERnd, the BG POWER increment was substantially greater than UG (Fig 2). In this regard, relative strength/power may be essential for COD performance (13). However, this relationship is not observed in our results.

## Conclusions

In summary, the results of our study show that 6 weeks of unilateral/bilateral eccentric overload training improved lower limb muscle volume, power, CMJ performance, COD90 and DEC-COD90, without changes in sprint performance in team sport athletes. This finding suggests that EO training provided a better COD turn of the 90° transition phase. Our results also show that UG improved COD90 and DEC-COD90 in the dominant and non-dominant legs, while BG only improved COD90 and DEC-COD90 in the dominant leg. This finding suggests that unilateral training seems to be more effective in improving COD turns of 90° performance.
